# Different Factors Associated with Morning Blood Pressure Surge in Antihypertensive-Naïve Dipper and Non-Dipper Subjects

**DOI:** 10.3390/jcm12072464

**Published:** 2023-03-23

**Authors:** Yi-Hsin Chu, Zih-Jie Sun, Yin-Fan Chang, Yi-Ching Yang, Chih-Jen Chang, Yu-Tsung Chou, Jin-Shang Wu

**Affiliations:** 1 Department of Family Medicine, National Cheng Kung University Hospital, College of Medicine, National Cheng Kung University, Tainan 70403, Taiwan; 2 Department of Family Medicine, National Cheng Kung University Hospital, Dou-Liou Branch, College of Medicine, National Cheng Kung University, Yunlin 64043, Taiwan; 3 Department of Family Medicine, College of Medicine, National Cheng Kung University, Tainan 70101, Taiwan; 4 Department of Family Medicine, Ditmanson Medical Foundation Chia-Yi Christian Hospital, Chiayi 60002, Taiwan; 5 Department of Health Management Center, National Cheng Kung University Hospital, College of Medicine, National Cheng Kung University, Tainan 70403, Taiwan

**Keywords:** ambulatory blood pressure monitoring, dipping blood pressure, parasympathetic withdrawal

## Abstract

The relationship between the morning blood pressure surge (MBPS) and cardiovascular risk is inconclusive. Previous studies have not taken into consideration dipping status in examining the MBPS and its associated factors. The aim was to examine factors associated with the MBPS in dippers and non-dippers. The MBPS was calculated by data obtained from ambulatory blood pressure monitoring, using the definition of sleep-trough morning surge. Dipping systolic blood pressure (DipSBP) was defined as [1 − (SBPsleeping/SBPawake)] × 100%. The value in milliseconds of standard deviation of normal-to-normal RR interval after waking up (SDNNaw) was calculated during the 2 h period after waking up. A total of 140 eligible subjects were divided into dippers (*n* = 62) and non-dippers (*n* = 78). Multiple regression analysis on data for all subjects revealed different correlations with the MBPS: positive in age, body mass index (BMI), and DipSBP, and inverse in cholesterol/high density lipoprotein-cholesterol (HDL-C) ratio, fasting blood glucose, and 2 h SDNNaw. When dippers were examined separately, age, female gender, and BMI correlated positively with MBPS, while cholesterol/HDL-C ratio and 2 h SDNNaw correlated negatively. For non-dippers, only age was associated with the MBPS. The factors associated with the MBPS were different for dippers and non-dippers. The MBPS seems to be a physiological response in this dipper group because age and BMI correlated positively with the MBPS, while parasympathetic neural activity after waking up and cholesterol/HDL-C ratio showed inverse correlations.

## 1. Introduction

The morning blood pressure surge (MBPS), which can be measured by means of ambulatory blood pressure monitoring (ABPM) [1], was first investigated in response to its possible involvement in the high occurrence rate of cardiovascular events in the morning [2,3,4]. Although some studies have shown a high MBPS to be associated with higher cardiovascular risk [5,6,7,8,9,10,11,12], others have revealed a neutral effect [13] or even a lower risk [14]. Such discrepancies have also been found in cross-sectional studies of the MBPS [15,16]. They may reflect differences in the definitions and cutoff values of the MBPS used in different studies [17], or they may be the result of effects related to age, the use of antihypertensive medications, or ethnicity [18].

It has been suggested that the response of the autonomic nervous system to activity performed after waking up, marked by an increase in sympathetic tone and a reciprocal decrease in parasympathetic tone, is the main mechanism underlying the MBPS [19,20,21,22,23]. Accordingly, based on the results of previous studies [14,24], we propose to investigate the following paradoxical tripartite relationships among the MBPS, nocturnal blood pressure (BP) dipping, and cardiovascular risk: (1) A non-dipping nocturnal BP pattern increases cardiovascular risk [25,26,27,28,29]. (2) A higher MBPS has also been associated with cardiovascular risk [5,6,7,8,9,10,11,12]. However, (3) the magnitude of the MBPS is inversely related to a non-dipping nocturnal BP pattern [14,30,31]. Logically speaking, these relationships cannot exist concurrently, and so the inherent paradox highlights the need to take the impact of nocturnal BP dipping into consideration when the MBPS is examined clinically because it may be associated with cardiovascular risk.

More recent studies [5,8,32] have shown that the MBPS may have different effects on cardiovascular risk in individuals who exhibit nocturnal BP dipping and in those who do not. For example, Pierdomenico et al. reported that a high MBPS predicted the occurrence of coronary events and strokes only in individuals with nocturnal BP dipping [5,8], with similar findings reported by Kario et al. in the Japan Ambulatory Blood Pressure Monitoring Prospective (JAMP) study [33]. The impact of nocturnal BP dipping was also highlighted in research by Gong et al., who reported that a relationship between an MBPS and subclinical target organ damage only existed in cases when nocturnal dipping occurred [34]. On the other hand, Israel et al. found a link between a high MBPS and lower mortality only in the presence of a non-dipping nocturnal BP pattern [32]. The results of the studies mentioned above inspired us to investigate the MBPS by dividing our subjects into groups based on whether or not they exhibited nocturnal BP dipping. In addition, although these studies have identified a relationship between the MBPS and cardiovascular risk factors such as age [35], the blood glucose level [15,16], the lipid profile [36], and the autonomic neural activity presenting with heart rate variability [31], none of them concomitantly took into consideration BP dipping status. Thus, the current study aimed to examine the factors associated with the MBPS in dipper and non-dipper subjects in order to clarify the possible role of the MBPS in cardiometabolic-related factors.

## 2. Materials and Methods

### 2.1. Participants

**Figure 1:**
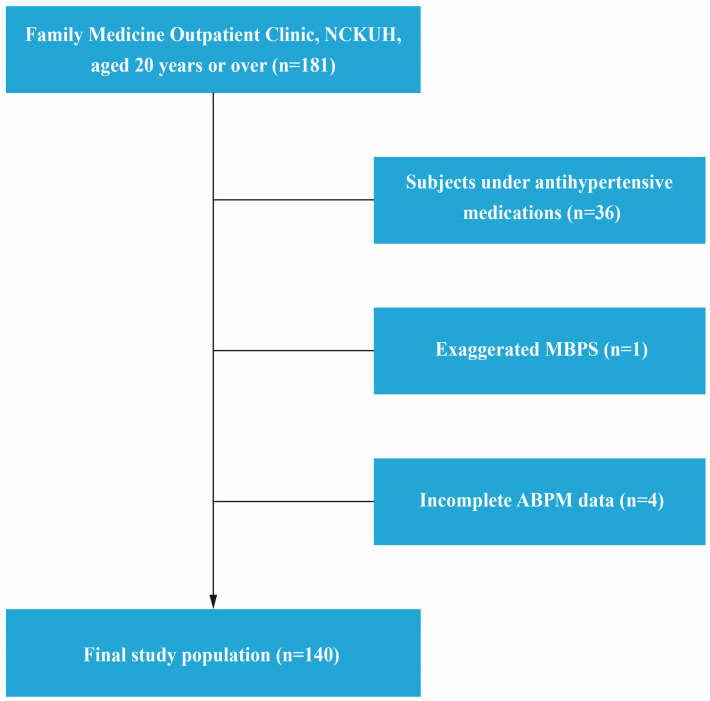
Flowchart of the process for determining inclusion in and exclusion from this study. ABPM, ambulatory blood pressure monitoring; MBPS, morning blood pressure surge; NCKUH, National Cheng Kung University Hospital.

The participants in this study were recruited from the group of patients aged ≥ 20 years who visited the outpatient clinic of the Department of Family Medicine at the National Cheng Kung University Hospital (NCKUH) between September 2010 and October 2011. Subjects who were taking antihypertensive medications (*n* = 36), exhibited an exaggerated MBPS (defined as MBPS value ≥ 55 mmHg, *n* = 1) [12], and had incomplete ABPM data (defined as less than 80% valid BP recordings, *n* = 4) were excluded (see Figure 1). The study protocol was approved by the Institutional Review Board of the NCKUH (ER-98-46 and ER-100-078).

### 2.2. Measurements

#### 2.2.1. Demographic and Basic Characteristics

Demographic characteristics, medical history, medication use, a family history of chronic diseases, alcohol use, smoking habits, and physical activity were assessed using a structured questionnaire performed by a well-trained assistant in a standardized process. Regular exercise was defined as exercising three times or more per week. At the outpatient clinic, subjects were instructed to be seated quietly for at least 5 min. After resting, BP was measured in the right upper-arm by mercury sphygmomanometer with the cuff bladder encircling at least 80% of the arm, based on the guideline of the Seventh Report of the Joint National Committee on Prevention, Detection, Evaluation, and Treatment of High Blood Pressure (JNC7) [37]. Taking off shoes and wearing light clothes, all subjects were measured for body weight (to the nearest 0.1 kg) and height (to the nearest 0.1 cm) by well-trained nurses using a certified machine. Their body mass index (BMI) was calculated by dividing their weight (in kilograms) by their height (in meters) squared. The biochemical parameters, including fasting blood glucose, cholesterol, high-density lipoprotein cholesterol (HDL-C), triglycerides, and creatinine, were measured after an overnight fast for at least 10 h.

#### 2.2.2. Ambulatory Blood Pressure Monitoring and Electrocardiography

Noninvasive 24 h ABPM (card(X)plore, Meditech Kft, Budapest, Hungary) was carried out by means of a device worn on the nondominant arm on a weekday and ABPM data was analyzed by its package software (CardioVisions). It was a validated device set used in a previous study [38]. The subjects were asked to perform their normal daily activities and to consume their habitual diet. The device was programmed to record their BP every 30 min for 24 h, at which times, the subjects were instructed to keep their arm still. All subjects were asked to record the exact times when they fell asleep and woke up. Only the BP recordings taken over the 24 h period with readings that were at least 80% valid were accepted. These recordings were then analyzed to obtain relevant ABPM data (as defined below). The nighttime BP was defined as the mean BP that was recorded from the time when the patients went to bed to the time when they woke up (sleeping time), and the daytime BP was defined as the mean BP during the remaining portion of the day (awake time). The dipping systolic blood pressure (DipSBP) was defined as [1 − (SBPsleeping/SBPawake)] × 100% [6]. A dipper subject was one whose DipSBP was ≥10%, and a non-dipper subject was one with a DipSBP of <10% [5]. The concept of the sleep-trough morning surge was the basis of the definition of the MBPS, which was calculated as the difference between the morning systolic BP (the average of the four 30-min systolic BP readings taken during the first 2 h after waking up) and the lowest nighttime systolic BP (the average of the three systolic BP readings centered on the lowest nighttime reading) [17].

Ambulatory electrocardiogram monitoring was used to record RR intervals. The standard deviation of the normal-to-normal RR interval (SDNN), an index of cardiac parasympathetic activity [39], was calculated after the deletion of any RR intervals that began or ended with a premature atrial or ventricular contraction. The value of the SDNN after waking up (SDNNaw) was calculated during the 2 h period after waking, indicated as the 2 h SDNNaw, and it was expressed in milliseconds in this study.

### 2.3. Statistical Analyses

The data analyses were performed using the Statistical Package for Social Sciences 22.0 software for Windows. The subjects were divided into two groups: the dippers and the non-dippers. In the univariate analysis, the independent sample *t*-test and the chi-square test were used to compare both groups in terms of the continuous variables and the categorical variables. The Mann–Whitney U-test was used to compare triglyceride level between groups due to non-Gaussian distribution. Square root transformation of triglyceride level was also performed for the independent sample t-test. A multiple linear regression analysis was performed to examine the independent associations between the clinical variables and the MBPS, as continuous variables, for all subjects, including both the dippers and the non-dippers. Default settings of backward selection was used for determining independent factors in the model. The beta coefficient and the 95% confidence interval of the clinical variables were estimated to determine their association with the MBPS. The adjusted R-square was calculated. A *p*-value of <0.05 was considered statistically significant. We also performed power analyses for this study given the study sample size (*n* = 140). Assuming a medium effect size for the association and a type 1 error of 0.05, a statistical power of 0.89 was expected for the regression model including 10 independent variables to predict the MBPS. The statistical power dramatically decreased to 0.38 when the association with the MBPS was examined for each of the 10 independent variables.

## 3. Results

A total of 140 eligible subjects were included in the final analysis. The clinical and biochemical characteristics of the dippers (*n* = 62) and the non-dippers (*n* = 78) are shown in Table 1. Compared to the dippers, the non-dippers had significantly higher values for age, nighttime systolic BP, and lowest nighttime systolic BP, and significantly lower values for DipSBP and MBPS. No significant differences were found between the groups for gender, BMI, outpatient systolic/diastolic BP, mean 24 h systolic/diastolic BP, mean daytime systolic BP, mean morning systolic BP, fasting blood glucose, cholesterol, triglycerides, HDL-C, creatinine, regular exercise, current alcohol use, and current smoking.

Table 2 shows the beta coefficient and the 95% confidence interval of the impact of clinical variables on the MBPS based on a multiple linear regression analysis including data for all subjects. The MBPS was positively associated with age (ꞵ = 0.324, 95% CI = 0.117 to 0.532, *p* = 0.003), BMI (ꞵ = 0.843, 95% CI = 0.238 to 1.449, *p* = 0.007), and the DipSBP (ꞵ = 0.872, 95% CI = 0.587 to 1.156, *p* < 0.001), and inversely associated with the cholesterol/HDL-C ratio (ꞵ = −2.477, 95% CI = −4.270 to −0.684, *p* = 0.007), fasting blood glucose (ꞵ = −0.079, 95% CI = −0.132 to −0.026, *p* = 0.004), and the 2 h SDNNaw (ꞵ = −0.106, 95% CI = −0.180 to −0.031, *p* = 0.006).

The results of the multiple linear regression analysis of the data for the subgroups of the dippers and non-dippers are shown in Table 3. In the case of the dippers, age (ꞵ = 0.463, 95% CI = 0.196 to 0.730, *p* = 0.001), female vs. male (ꞵ = 6.469, 95% CI = 0.841 to 12.098, *p* = 0.025), and BMI (ꞵ = 1.827, 95% CI = 0.784 to 2.871, *p* = 0.001) were positively correlated with the MBPS, while the cholesterol/HDL-C ratio (ꞵ = −4.459, 95% CI = −7.685 to −1.233, *p* = 0.008) and the 2 h SDNNaw (ꞵ = −0.150, 95% CI = −0.237 to −0.063, *p* = 0.001) were negatively associated with the MBPS. The statistical significance was borderline for the correlation between fasting plasma glucose (ꞵ = −0.068, 95% CI = −0.142 to 0.005, *p* = 0.067) and the DipSBP (ꞵ = 0.861, 95% CI = −0.099 to 1.821, *p* = 0.078) and the MBPS. For the non-dippers, on the other hand, only age (ꞵ = 0.293, 95% CI = 0.037 to 0.550, *p* = 0.026) was positively correlated with the MBPS. Although the inverse correlation between fasting plasma glucose (ꞵ = −0.065, 95% CI = −0.138 to 0.008, *p* = 0.080) and the MBPS was borderline, it did not reach significance.

## 4. Discussion

In this study, fasting blood glucose and the cholesterol/HDL-C ratio were the factors which were negatively associated with the MBPS for all subjects. The subgroup analysis also revealed the same relationship between the cholesterol/HDL-C ratio and the MBPS for the dippers. Given that a high level of blood sugar and a high cholesterol/HDL-C ratio have been shown to be positively correlated with both metabolic and cardiovascular risks [40,41], the inverse correlation found in the current study between the MBPS and both fasting blood glucose and the cholesterol/HDL-C ratio suggests that a high MBPS does not pose a cardiometabolic risk. In addition, the dippers in this study showed a positive correlation between both age and BMI and the MBPS. As dippers get older and their BMI increases, it may be possible for them to maintain an appropriate BP after waking up if they don’t exhibit impaired autonomic neural activity, given that increased sympathetic neural activity is a hallmark of aging and obesity [42]. Furthermore, the 2 h SDNNaw, an index of parasympathetic neural activity, was also found to be inversely correlated with the MBPS for the dippers in the current study, which is compatible with the previous finding of a decrease in parasympathetic neural activity after people wake up [43].

On the basis of the correlations between certain factors and the MBPS mentioned above, which were obtained in the current study (positive for age and BMI and inverse for the 2 h SDNNaw and the cholesterol/HDL-C ratio), the MBPS does not seem to present a cardiometabolic risk and, in fact, may reflect a physiological response by the dippers in this study. This result contradicts a number of previous studies that have found a high MBPS to be associated with a high cardiovascular risk [5,6,7,8,9,10,11,12]. A possible explanation may be that the MBPS obtained in the current study ranged between −11.6 mmHg and 54.5 mmHg, after one subject was excluded for having a pathologically exaggerated MBPS of ≥ 55 mmHg, which was determined as the cutoff based on a previous study [12]. Thus, our findings may represent one part of the complete picture of the impact of the MBPS, and more studies are needed to further explore the physiological and pathological roles of the MBPS.

It has been shown that the autonomic nervous system plays an important role in the regulation of the circadian rhythm which the BP follows [22,23]. In addition, the MBPS has been shown to correlate with sympathetic reactivity, which was determined by recording the sensitivity of muscle sympathetic nerve activity to a cold pressor test [19]. The current study showed a negative association between the MBPS and the 2 h SDNNaw, which is compatible with the findings of a previous study [31] highlighting the reciprocal relationship between sympathetic and parasympathetic neural activity [44]. Our finding of a relationship between the MBPS and the 2 h SDNNaw is also consistent with previous studies, which have suggested that the time just after waking up plays an important role in the MBPS through the intermediary of either neurohormonal modulation [22] or physical activity [45]. Finally, it stands to reason that we found a correlation between the MBPS and the DipSBP, which is well-documented in the literature [14,30], since the calculation of the MBPS is largely related to the daytime BP and the nighttime BP.

This study found a positive association between the MBPS and both age and BMI. It has previously been found that increased sympathetic neural activity is a consequence of both aging and obesity [42], and it may, therefore, exert a considerable influence on the circadian rhythm followed by the BP. A positive association between age and the MBPS has previously been found, with an age-related increase in arterial stiffness and endothelial dysfunction posited as the possible mechanism involved [35]. Recently, the augmented venoarteriolar response in the lower limbs of elderly subjects has been suggested as a possible link between aging and the MBPS [46]. However, no published paper so far has established a direct link between the MBPS and BMI. The correlation between the MBPS with the waist-hip ratio [47] and the oxygen desaturation in obstructive sleep apnea patients [48] suggests a possible interaction between the MBPS and obesity, although the exact mechanism underlying this correlation is still unknown.

In the current study, we found the female subjects exhibited a higher MBPS than the male subjects after adjusting for the other clinical variables for the dippers, but not for the non-dippers. Similarly, in a recent study, no significant difference in the MBPS was found between young female subjects (mean age: 24.4 ± 4.5 years) and young male subjects (mean age: 25.5 ± 6.3 years) at a low altitude (400 m above sea level), but the female MBPS (16.1 ± 13.0 mmHg) was higher than that of males (6.36 ± 11.1 mmHg) at a high altitude (4100 m above sea level) [49]. It has been suggested that this gender difference can be attributed to women experiencing a greater parasympathetic withdrawal compared to men during the task performance [50]. However, we adjusted for the 2 h SDNNaw in our multivariate analysis, which suggests that factors other than parasympathetic withdrawal are behind the positive association between females and the MBPS. One possible explanation is that women are more likely to have anxiety [51], given that anxiety has been shown to correlate with awake systolic BP in women [52]. Another factor that may play a role in the MBPS of women is renin. Lower levels of renin and aldosterone have been found in subjects who exhibited an MBPS compared to those who did not [53], and women seem to have a lower level of renin than men [54,55]. Further research is warranted in order to determine what mechanisms underly the positive correlation between being a woman and experiencing an MBPS.

In the current study, we found that the cholesterol/HDL-C ratio and fasting blood glucose were negatively associated with the MBPS for all subjects. This is surprising given that these are well-established cardiovascular risk factors. For instance, one previous study showed that the MBPS was positively associated with low-density lipoprotein (LDL) cholesterol [36]. Our contradictory finding may be related to our method of selecting our subjects, differences in BP dipping status, exaggerated MBPSs, or other unknown factors. Furthermore, while we found a clear negative association between the MBPS and fasting blood glucose for all subjects, the correlation was borderline when the dippers and non-dippers were examined separately. Other studies have also produced results that contrasted with ours. For example, Yoda et al. reported a positive association between the MBPS and the level of HbA1c for subjects with type 2 diabetes mellitus whose mean age was 60.1 ± 13.2 years, who had had diabetes for an average of 9 years, and who had a high mean level of HbA1c (8.7 ± 1.4%) [16]. Shimizu et al. also reported a positive association between the MBPS and fasting blood glucose for hypertensive subjects who were elderly (mean age: 72.2 ± 8.5 years) and had high clinical measurements of systolic BP (164 ± 19 mmHg) [56]. Under normal condition, dippers show an appropriate parasympathetic withdrawal and sympathetic activation. In contrast, non-dippers may exhibit varying degrees of autonomic dysfunction, characterized by less parasympathetic withdrawal and more sympathetic activation, in order to maintain their BP after waking up [57,58]. A possible explanation for the difference in findings between our study and those carried out by Yoda et al. and Shimizu et al. is related to the type of subjects that were examined—a mixed population in our study, diabetic subjects with a high HbA1c (8.7 ± 1.4%) in the study by Yoda et al., and hypertensive elderly subjects with a high systolic BP (164 ± 19 mmHg) in the study by Shimizu et al. It is likely that diabetic and hypertensive populations contain a higher proportion of non-dippers [59,60] who may exhibit exaggerated sympathetic activity and reduced parasympathetic activity, resulting in a positive relationship between blood sugar levels and the MBPS.

Our study had several limitations. First, the cross-sectional design limited our ability to identify causal relationships. Second, the ABPM data were recorded during a single 24 h period. Despite previous studies arguing in support of the reproducibility of APBM data [17], only moderate reproducibility has been found in conjunction with the sleep-trough morning surge, which we used, and remains one of the most widely used definition of the MBPS [61]. Third, the factors associated with sleep apnea, such as age, male gender, BMI, high blood pressure, hyperglycemia, and dyslipidemia [62,63,64], were collected and adjusted in this study. The confounding effect of sleep apnea was not completely ruled out. Fourth, nonsignificant correlations in the linear regression models, especially in the non-dipper group, might be caused by the relatively small sample size. Analysis with a larger sample size may be performed in the future. Finally, the current study was carried out with Taiwanese subjects, and so the results may not be generalizable to other ethnic populations.

## 5. Conclusions

In conclusion, the MBPS associated positively with age, female gender, and BMI, and negatively with cholesterol/HDL-C ratio and 2 h SDNNaw in the dipper group. Only age was the positively associated factor of the MBPS in the non-dipper group. The main takeaway from our study is that it highlights the factors that contribute to differences between dippers and non-dippers in relation to the MBPS. Furthermore, the MBPS seems to be a physiological response in our dipper population based on the findings that age and BMI were the positively correlated factors and parasympathetic activity after waking up and the cholesterol/HDL-C ratio were the inversely correlated factors. The results provide a direction for future research into the pathological and physiological roles of the MBPS in cardiometabolic-related factors.

## Figures and Tables

**Table 1:** Clinical characteristics of dippers and non-dippers.

	Dippers (*n* = 62)	Non-Dippers (*n* = 78)	*p*-Value
Age, in years	52.2 ± 11.8	56.2 ± 10.9	0.041
Female (%)	23 (37.1)	34 (43.6)	0.437
BMI, kg/m^2^	24.9 ± 3.9	25.4 ± 4.3	0.496
Outpatient systolic BP, mmHg	142.1 ± 18.6	143.6 ± 15.4	0.584
Outpatient diastolic BP, mmHg	89.3 ± 13.0	89.0 ± 11.6	0.872
24 h mean systolic BP, mmHg	131.2 ± 13.5	133.8 ± 17.0	0.320
24 h mean diastolic BP, mmHg	80.2 ± 9.8	79.6 ± 10.8	0.720
Daytime systolic BP, mmHg	138.1 ± 14.2	135.6 ± 17.7	0.373
Nighttime systolic BP, mmHg	118.4 ± 13.2	130.6 ± 17.1	<0.001
Dipping systolic blood pressure, %	14.3 ± 3.0	3.63 ± 4.9	<0.001
Morning systolic BP, mmHg	137.8 ± 16.9	137.4 ± 19.8	0.892
Lowest nighttime systolic BP, mmHg	110.9 ± 13.4	122.4 ± 18.1	<0.001
Morning blood pressure surge, mmHg	26.9 ± 12.6	15.9 ± 10.7	<0.001
2 h SDNNaw, ms	79.2 ± 31.1	79.0 ± 22.3	0.967
Fasting blood glucose, mg/dL	113.1 ± 37.8	116.4 ± 40.2	0.633
Cholesterol/HDL-C ratio	3.70 ± 1.17	3.90 ± 1.36	0.393
Cholesterol, mg/dL	191.1 ± 38.6	201.1 ± 41.3	0.151
HDL-C, mg/dL	56.1 ± 15.9	56.6 ± 19.4	0.887
Triglycerides, mg/dL	146.3 ± 122.7	121.9 ± 104.4	0.278 *
Square root of triglycerides, $\sqrt{\mathrm{mg}/\mathrm{dL}}$	11.4 ± 4.1	10.6 ± 3.2	0.191
Creatinine, mg/dL	0.83 ± 0.17	0.82 ± 0.16	0.766
Current alcohol use	11 (17.7)	21 (26.9)	0.199
Current smoking	19 (30.6)	19 (24.4)	0.406
Regular exercise	22 (35.5)	38 (48.7)	0.116

Data expressed as mean ± standard deviation or number (=percentage); BMI, body mass index; BP, blood pressure; SDNNaw, standard deviation of normal-to-normal RR interval after waking up; HDL-C, high-density lipoprotein-cholesterol. * Mann–Whitney U-test. Others as independent sample *t*-tests or the chi-square tests.

**Table 2:** Beta coefficient and 95% confidence interval of clinical variables on impact of morning blood pressure surge based on multiple linear regression analysis.

	Total Subjects (*n* = 140)		
	ꞵ Coefficient	95% CI	*p*-Value
Age, in years	0.324	0.117~0.532	0.003
Female vs. male	3.975	−0.691~8.641	0.094
BMI, kg/m^2^	0.843	0.238~1.449	0.007
Cholesterol/HDL-C ratio	−2.477	−4.270~−0.684	0.007
Fasting blood glucose, mg/dL	−0.079	−0.132~−0.026	0.004
2 h SDNNaw, ms	−0.106	−0.180~−0.031	0.006
Dipping systolic blood pressure, %	0.872	0.587~1.156	<0.001
Current alcohol use, %	2.332	−3.111~7.774	0.398
Current smoking, %	4.078	−1.360~9.516	0.140
Regular exercise, %	2.301	−2.124~6.726	0.305

Adjusted R-square = 35.6%; CI, confidence interval; BMI, body mass index; HDL-C, high-density lipoprotein-cholesterol; SDNNaw, standard deviation of normal-to-normal RR interval after waking up.

**Table 3:** Beta coefficient and 95% confidence interval of clinical variables on impact of morning blood pressure surge based on multiple linear regression analysis comparing dippers and non-dippers.

	Dippers (*n* = 62)			Non-Dippers (*n* = 78)		
	Beta Coefficient	95% CI	*p*-Value	Beta Coefficient	95% CI	*p*-Value
Age, in years	0.463	0.196~0.730	0.001	0.293	0.037~0.550	0.026
Female vs. male	6.469	0.841~12.098	0.025	−2.948	−8.485~2.589	0.291
BMI, kg/m^2^	1.827	0.784~2.871	0.001	0.535	−0.216~1.286	0.159
Cholesterol/HDL-C ratio	−4.459	−7.685~−1.233	0.008	−1.502	−3.708~0.705	0.178
Fasting blood glucose, mg/dL	−0.068	−0.142~0.005	0.067	−0.065	−0.138~0.008	0.080
2 h SDNNaw, ms	−0.150	−0.237~−0.063	0.001	−0.074	−0.193~0.044	0.215
Dipping systolic blood pressure, %	0.861	−0.099~1.821	0.078	0.349	−0.205~0.902	0.231

Adjusted R-square = 45.0% (dippers), 10.7% (non-dippers); CI, confidence interval; BMI, body mass index; HDL-C, high-density lipoprotein-cholesterol; SDNNaw, standard deviation of normal-to-normal RR interval after waking up.

## Data Availability

The data presented in this study are available on request from the corresponding author. The data are not publicly available due to privacy restriction.

## References

[1] Giles T.D. (2006). Circadian rhythm of blood pressure and the relation to cardiovascular events. J. Hypertens..

[2] Elliott W.J. (1998). Circadian variation in the timing of stroke onset: A meta-analysis. Stroke.

[3] Muller J.E., Tofler G.H., Stone P.H. (1989). Circadian variation and triggers of onset of acute cardiovascular disease. Circulation.

[4] Muller J.E., Stone P.H., Turi Z.G., Rutherford J.D., Czeisler C.A., Parker C., Poole W.K., Passamani E., Roberts R., Robertson T. (1985). Circadian variation in the frequency of onset of acute myocardial infarction. N. Engl. J. Med..

[5] Pierdomenico S.D., Pierdomenico A.M., Di Tommaso R., Coccina F., Di Carlo S., Porreca E., Cuccurullo F. (2016). Morning blood pressure surge, dipping, and risk of coronary events in elderly treated hypertensive patients. Am. J. Hypertens..

[6] Pierdomenico S.D., Pierdomenico A.M., Coccina F., Lapenna D., Porreca E. (2016). Circadian blood pressure changes and cardiovascular risk in elderly-treated hypertensive patients. Hypertens. Res..

[7] Turak O., Afsar B., Ozcan F., Canpolat U., Grbovic E., Mendi M.A., Oksuz F., Siriopol D., Covic A., Caliskan M. (2014). Relationship Between Elevated Morning Blood Pressure Surge, Uric Acid, and Cardiovascular Outcomes in Hypertensive Patients. J. Clin. Hypertens..

[8] Pierdomenico S.D., Pierdomenico A.M., Cuccurullo F. (2014). Morning blood pressure surge, dipping, and risk of ischemic stroke in elderly patients treated for hypertension. Am. J. Hypertens..

[9] Shimizu M., Ishikawa J., Yano Y., Hoshide S., Shimada K., Kario K. (2011). The relationship between the morning blood pressure surge and low-grade inflammation on silent cerebral infarct and clinical stroke events. Atherosclerosis.

[10] Kario K., Yano Y., Matsuo T., Hoshide S., Eguchi K., Shimada K. (2011). Additional impact of morning haemostatic risk factors and morning blood pressure surge on stroke risk in older Japanese hypertensive patients. Eur. Heart J..

[11] Li Y., Thijs L., Hansen T.W., Kikuya M., Boggia J., Richart T., Metoki H., Ohkubo T., Torp-Pedersen C., Kuznetsova T. (2010). Prognostic value of the morning blood pressure surge in 5645 subjects from 8 populations. Hypertension.

[12] Kario K., Pickering T.G., Umeda Y., Hoshide S., Hoshide Y., Morinari M., Murata M., Kuroda T., Schwartz J.E., Shimada K. (2003). Morning surge in blood pressure as a predictor of silent and clinical cerebrovascular disease in elderly hypertensives: A prospective study. Circulation.

[13] Bombelli M., Fodri D., Toso E., Macchiarulo M., Cairo M., Facchetti R., Dell’Oro R., Grassi G., Mancia G. (2014). Relationship among morning blood pressure surge, 24-hour blood pressure variability, and cardiovascular outcomes in a white population. Hypertension.

[14] Verdecchia P., Angeli F., Mazzotta G., Garofoli M., Ramundo E., Gentile G., Ambrosio G., Reboldi G. (2012). Day-night dip and early-morning surge in blood pressure in hypertension: Prognostic implications. Hypertension.

[15] Lyhne J.M., Laugesen E., Høyem P., Cichosz S., Christiansen J.S., Knudsen S.T., Hansen K.W., Hansen T.K., Poulsen P.L. (2015). Morning blood pressure surge and target organ damage in newly diagnosed type 2 diabetic patients: A cross sectional study. BMC Endocr. Disord..

[16] Yoda K., Inaba M., Hamamoto K., Yoda M., Tsuda A., Mori K., Yamada S., Emoto M., Koyama H., Imanishi Y. (2014). Association between glycemic control and morning blood pressure surge with vascular endothelial dysfunction in type 2 diabetic patients. Diabetes Care.

[17] Kario K. (2010). Morning surge in blood pressure and cardiovascular risk: Evidence and perspectives. Hypertension.

[18] Kario K. (2015). Prognosys in Relation to Blood Pressure Variability. Hypertension.

[19] Lambert E.A., Chatzivlastou K., Schlaich M., Lambert G., Head G.A. (2014). Morning surge in blood pressure is associated with reactivity of the sympathetic nervous system. Am. J. Hypertens..

[20] Grassi G., Bombelli M., Seravalle G., Dell’Oro R., Quarti-Trevano F. (2010). Diurnal blood pressure variation and sympathetic activity. Hypertens. Res..

[21] Kario K., Pickering T.G., Hoshide S., Eguchi K., Ishikawa J., Morinari M., Hoshide Y., Shimada K. (2004). Morning blood pressure surge and hypertensive cerebrovascular disease: Role of the alpha adrenergic sympathetic nervous system. Am. J. Hypertens..

[22] Dodt C., Breckling U., Derad I., Fehm H.L., Born J. (1997). Plasma epinephrine and norepinephrine concentrations of healthy humans associated with nighttime sleep and morning arousal. Hypertension.

[23] Panza J.A., Epstein S.E., Quyyumi A.A. (1991). Circadian variation in vascular tone and its relation to alpha-sympathetic vasoconstrictor activity. N. Engl. J. Med..

[24] Sheppard J.P., Hodgkinson J., Riley R., Martin U., Bayliss S., McManus R.J. (2015). Prognostic significance of the morning blood pressure surge in clinical practice: A systematic review. Am. J. Hypertens..

[25] Hansen T.W., Li Y., Boggia J., Thijs L., Richart T., Staessen J.A. (2011). Predictive role of the nighttime blood pressure. Hypertension.

[26] Fagard R.H., Celis H., Thijs L., Staessen J.A., Clement D.L., De Buyzere M.L., De Bacquer D.A. (2008). Daytime and nighttime blood pressure as predictors of death and cause-specific cardiovascular events in hypertension. Hypertension.

[27] Verdecchia P. (2000). Prognostic Value of Ambulatory Blood Pressure. Hypertension.

[28] Staessen J.A., Thijs L., Fagard R., O’Brien E.T., Clement D., de Leeuw P.W., Mancia G., Nachev C., Palatini P., Parati G. (1999). Predicting Cardiovascular Risk Using Conventional vs. Ambulatory Blood Pressure in Older Patients With Systolic Hypertension. JAMA.

[29] Verdecchia P., Porcellati C., Schillaci G., Borgioni C., Ciucci A., Battistelli M., Guerrieri M., Gatteschi C., Zampi I., Santucci A. (1994). Ambulatory blood pressure. An independent predictor of prognosis in essential hypertension. Hypertension.

[30] Metoki H., Ohkubo T., Kikuya M., Asayama K., Obara T., Hashimoto J., Totsune K., Hoshi H., Satoh H., Imai Y. (2006). Prognostic significance for stroke of a morning pressor surge and a nocturnal blood pressure decline: The Ohasama study. Hypertension.

[31] Mokwatsi G.G., Schutte A.E., Mels C.M.C., Kruger R. (2019). Morning Blood Pressure Surge Relates to Autonomic Neural Activity in Young Non-Dipping Adults: The African-PREDICT Study. Heart Lung Circ..

[32] Israel S., Israel A., Ben-Dov I.Z., Bursztyn M. (2011). The morning blood pressure surge and all-cause mortality in patients referred for ambulatory blood pressure monitoring. Am. J. Hypertens..

[33] Hoshide S., Kario K. (2021). Morning Surge in Blood Pressure and Stroke Events in a Large Modern Ambulatory Blood Pressure Monitoring Cohort: Results of the JAMP Study. Hypertension.

[34] Gong S., Liu K., Ye R., Li J., Yang C., Chen X. (2019). Nocturnal dipping status and the association of morning blood pressure surge with subclinical target organ damage in untreated hypertensives. J. Clin. Hypertens..

[35] Lee D.H., Ihm S.H., Youn H.J., Choi Y.S., Park C.S., Park C.S., Lee J.M., Kim H.Y., Oh Y.S., Chung W.S. (2009). Age is an independent risk factor for the early morning blood pressure surge in patients never-treated for hypertension. Korean Circ. J..

[36] Martin C.A., Cameron J.D., Head G.A., Chen S.S., Eikelis N., McGrath B.P. (2013). The morning blood pressure surge is related to serum cholesterol. J. Hum. Hypertens..

[37] Chobanian A.V., Bakris G.L., Black H.R., Cushman W.C., Green L.A., Izzo J.L., Jones D.W., Materson B.J., Oparil S., Wright J.T. (2003). Seventh report of the Joint National Committee on Prevention, Detection, Evaluation, and Treatment of High Blood Pressure. Hypertension.

[38] Currie K.D., Hubli M., MacDonald M.J., Krassioukov A.V. (2019). Associations between arterial stiffness and blood pressure fluctuations after spinal cord injury. Spinal Cord.

[39] Yilmaz M., Kayancicek H., Cekici Y. (2018). Heart rate variability: Highlights from hidden signals. J. Integr. Cardiol..

[40] Holman R.R., Paul S.K., Bethel M.A., Matthews D.R., Neil H.A.W. (2008). 10-Year Follow-up of Intensive Glucose Control in Type 2 Diabetes. N. Engl. J. Med..

[41] Stamler J., Wentworth D., Neaton J.D. (1986). Is relationship between serum cholesterol and risk of premature death from coronary heart disease continuous and graded? Findings in 356,222 primary screenees of the Multiple Risk Factor Intervention Trial (MRFIT). JAMA.

[42] Balasubramanian P., Hall D., Subramanian M. (2019). Sympathetic nervous system as a target for aging and obesity-related cardiovascular diseases. Geroscience.

[43] Huikuri H.V., Kessler K.M., Terracall E., Castellanos A., Linnaluoto M.K., Myerburg R.J. (1990). Reproducibility and circadian rhythm of heart rate variability in healthy subjects. Am. J. Cardiol..

[44] Shaffer F., Ginsberg J.P. (2017). An Overview of Heart Rate Variability Metrics and Norms. Front. Public Health.

[45] Leary A.C., Struthers A.D., Donnan P.T., MacDonald T.M., Murphy M.B. (2002). The morning surge in blood pressure and heart rate is dependent on levels of physical activity after waking. J. Hypertens..

[46] Yoo J.K., Sun D.D., Parker R.S., Urey M.A., Romero S.A., Lawley J.S., Sarma S., Vongpatanasin W., Crandall C.G., Fu Q. (2018). Augmented venoarteriolar response with ageing is associated with morning blood pressure surge. Exp. Physiol..

[47] Feldstein C.A., Akopian M., Olivieri A.O., Kramer A.P., Nasi M., Garrido D. (2005). A comparison of body mass index and waist-to-hip ratio as indicators of hypertension risk in an urban Argentine population: A hospital-based study. Nutr. Metab. Cardiovasc. Dis..

[48] Cho J.S., Ihm S.H., Kim C.J., Park M.W., Her S.H., Park G.M., Kim T.S. (2015). Obstructive Sleep Apnea Using Watch-PAT 200 Is Independently Associated With an Increase in Morning Blood Pressure Surge in Never-Treated Hypertensive Patients. J. Clin. Hypertens..

[49] Chen R., Yang J., Liu C., Sun M., Ke J., Yang Y., Shen Y., Yuan F., He C., Cheng R. (2021). Sex-Dependent Association Between Early Morning Ambulatory Blood Pressure Variations and Acute Mountain Sickness. Front. Physiol..

[50] Nugent A.C., Bain E.E., Thayer J.F., Sollers J.J., Drevets W.C. (2011). Sex differences in the neural correlates of autonomic arousal: A pilot PET study. Int. J. Psychophysiol..

[51] Wittchen H.-U., Zhao S., Kessler R.C., Eaton W.W. (1994). DSM-III-R Generalized Anxiety Disorder in the National Comorbidity Survey. Arch. Gen. Psychiatry.

[52] Kario K., Schwartz J.E., Davidson K.W., Pickering T.G. (2001). Gender differences in associations of diurnal blood pressure variation, awake physical activity, and sleep quality with negative affect: The work site blood pressure study. Hypertension.

[53] Cho J.S., Ihm S.H., Jang S.W., Chung W.B., Choi Y.S., Shin D.I., Seo S.M., Park M.W., Kim G.H., Her S.H. (2014). Negative association between plasma aldosterone concentration/plasma renin activity and morning blood pressure surge in never-treated hypertensive patients. Clin. Exp. Hypertens..

[54] Reckelhoff J.F. (2001). Gender Differences in the Regulation of Blood Pressure. Hypertension.

[55] Schunkert H., Jan Danser A.H., Hense H.W., Derkx F.H.M., Kürzinger S., Riegger G.A.J. (1997). Effects of estrogen replacement therapy on the renin-angiotensin system in postmenopausal women. Circulation.

[56] Shimizu M., Ishikawa J., Eguchi K., Hoshide S., Shimada K., Kario K. (2009). Association of an abnormal blood glucose level and morning blood pressure surge in elderly subjects with hypertension. Am. J. Hypertens..

[57] Cilsal E. (2020). In newly diagnosed hypertensive children, increased arterial stiffness and reduced heart rate variability were associated with a non-dipping blood pressure pattern. Rev. Port. Cardiol..

[58] Kawamura H., Ozawa Y., Izumi Y., Kasamaki Y., Nakayama T., Mitsubayashi H., Ohta M., Ichimaru Y. (2016). Non-dipping blood pressure variations in adult Kazakhs are derived from decreased daytime physical activity and increased nighttime sympathetic activity. Clin. Exp. Hypertens..

[59] Zhang J., Wang C., Gong W., Ye Z., Tang Y., Zhao W., Peng H., Lou T. (2017). Poor sleep quality is responsible for the nondipper pattern in hypertensive but not in normotensive chronic kidney disease patients. Nephrology.

[60] Ayala D.E., Moya A., Crespo J.J., Castineira C., Dominguez-Sardina M., Gomara S., Sineiro E., Mojon A., Fontao M.J., Hermida R.C. (2013). Circadian pattern of ambulatory blood pressure in hypertensive patients with and without type 2 diabetes. Chronobiol. Int..

[61] Stergiou G.S., Mastorantonakis S.E., Roussias L.G. (2008). Morning Blood Pressure Surge: The Reliability of Different Definitions. Hypertens. Res..

[62] Nadeem R., Singh M., Nida M., Waheed I., Khan A., Ahmed S., Naseem J., Champeau D. (2014). Effect of obstructive sleep apnea hypopnea syndrome on lipid profile: A meta-regression analysis. J. Clin. Sleep Med..

[63] Jennum P., Riha R.L. (2009). Epidemiology of sleep apnoea/hypopnoea syndrome and sleep-disordered breathing. Eur. Respir. J..

[64] Young T., Skatrud J., Peppard P.E. (2004). Risk factors for obstructive sleep apnea in adults. JAMA.

